# Supplementary material for: Long-term effects of working memory retrieval from prioritized and deprioritized states
Source: Commun Psychol. 2026 Jan 28;4:32. doi: 10.1038/s44271-026-00399-7 (PMC12913606; doi:10.1038/s44271-026-00399-7)
Supplement: Supplementary file 2 — Supplementary Material [file 44271_2026_399_MOESM2_ESM.pdf]

Supplementary Results

Supplementary Figure 1

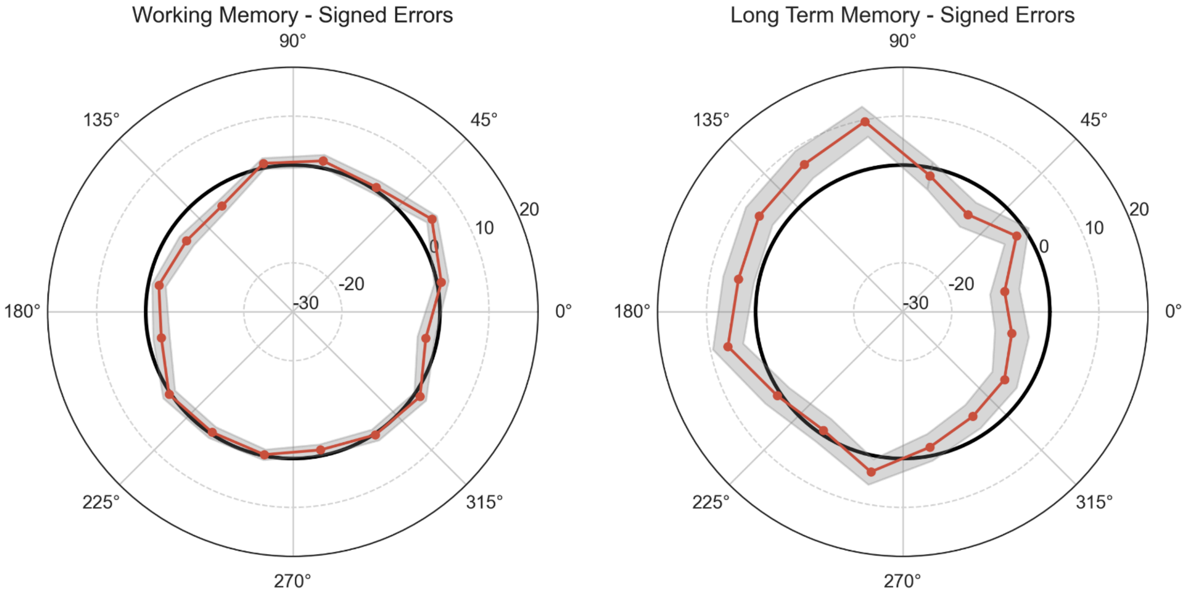

Supplementary Figure 1. Exploring cardinal repulsion bias in WM and LTM reports. An alternative explanation for our finding that participants' LTM reports were biased towards their previous WM reports (Fig. 2c) could be that both reports (WM and LTM) exhibited canonical "cardinal" bias. (Repulsive) cardinal bias (Bae, 2021; Taylor & Bays, 2018) refers to the finding that behavioral reports of stimulus orientation (for example, of Gabor gratings) can be biased away from the cardinal (vertical and horizontal) axes. In our present experiments, such a phenomenon can only be examined for a subset of stimulus objects (n = 63) which had a clear real-world upright position (see examples in Fig. 1; other objects, such as scissors were excluded). The polar plots show the mean signed error in degrees (cw < 0 < ccw) for each sample orientation in Exp.1, where 90° refers to the objects' upright orientation. Cardinal repulsion bias would be evident if the response errors were consistently positive (ccw) to the ccw side, and negative (cw) to the cw side of the cardinal axes (0°, 90°, 180°, and 360°; cf. Linde-Domingo & Spitzer, 2024). However, there was no clear indication of such systematic patterns in the present data, and the patterns in the WM (*left*) and LTM (*right*) tests were dissimilar (if anything, the mean signed errors correlated even negatively, $r_{Spearman} = -0.41$). Our findings in Fig. 2c can thus not be easily explained in terms of canonical cardinal bias.

Supplementary Figure 2

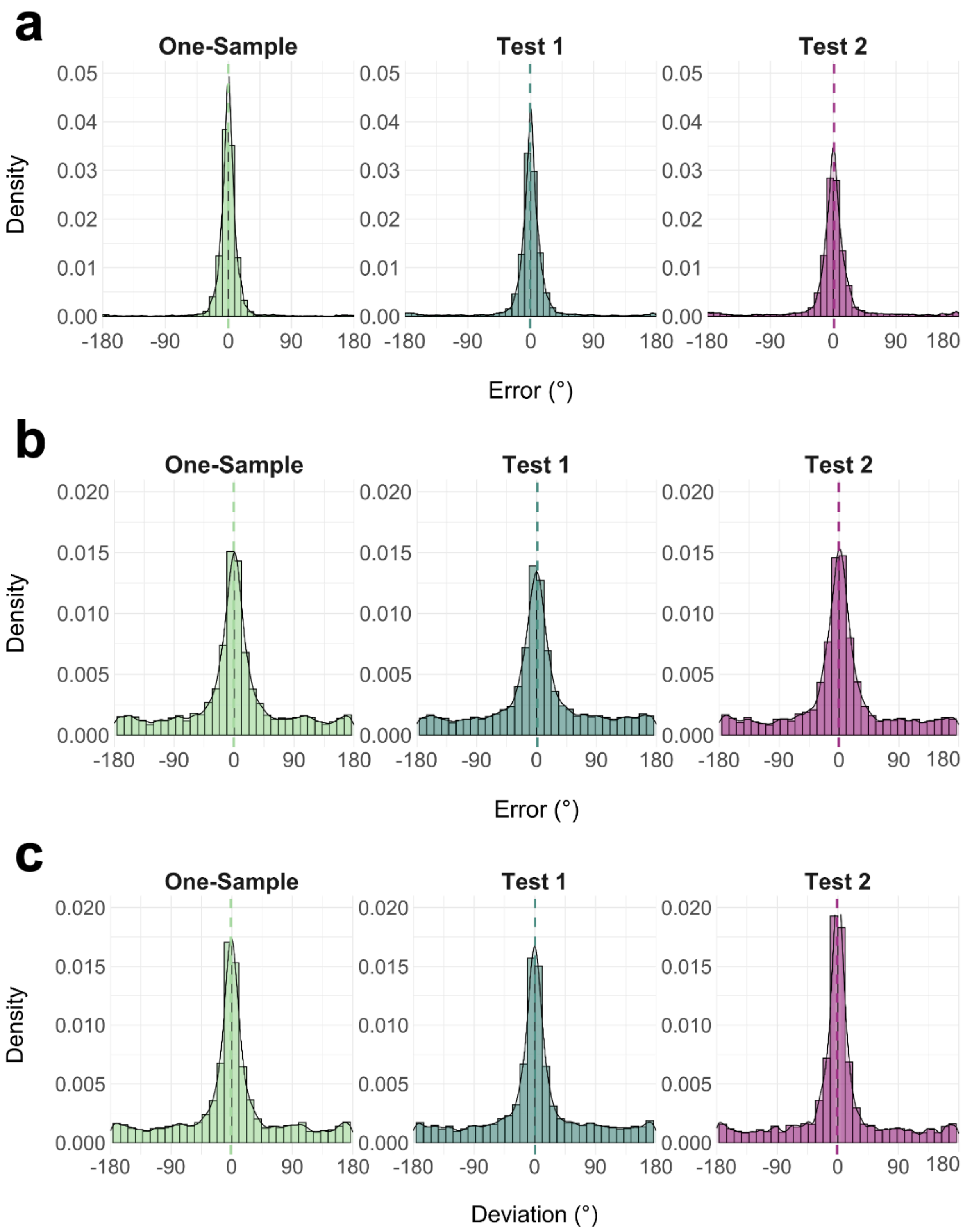

Supplementary Figure 2. Error distributions in the WM and LTM tests in Exp. 1. a, WM-test errors **b,** LTM-test errors (cf. Fig. 2c, dashed) **c,** LTM deviations from WM reports (cf. Fig. 2c, dark blue).

Supplementary Figure 3

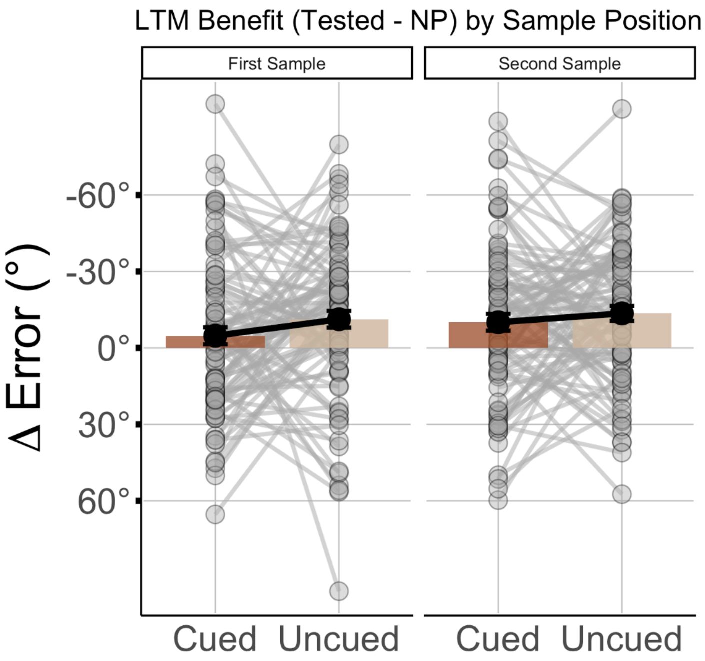

Supplementary Figure 3. No interaction of WM-testing benefit with sample position. Same as Fig. 3d, but plotted separately for WM samples presented first (left) or second (right). There was no significant interaction with the samples' presentation order (see main text).

Supplementary Analysis 1

Inter-item repulsive bias on two-sample trials. As another potential source of bias in both WM and LTM tests, upon reviewer suggestion, we examined if there was crosstalk between the two sample orientations presented on the same WM trial (two-sample trials in Exp. 1). To this end, we computed the absolute difference between participants' orientation reports and the orientation of the respective other item on the trial. Under the null-hypothesis of no inter-item bias, we would expect an orientation difference at chance-level (90°). Mean values < 90° would indicate inter-item attraction (resp. occasional confusion of the two orientations), and values > 90° would indicate repulsion (reporting the orientations to be more dissimilar from each other than they actually were; for related findings see (Kang et al., 2011). We found evidence for the latter, both in WM (Test 1: mean = 93.92°, SE = 0.64, Test 2: mean = 93.50°, SE = 0.78, both p < 0.001) and LTM reports (Test 1: mean = 92.56°, SE = 0.59, Test 2: mean = 93.70°, both p < 0.001). Importantly, however, this effect did not differ between the Test 1 and Test 2 conditions (prioritized/deprioritized), neither in WM [t(186) = 0.600, p = 0.549, *d* = 0.0438] nor LTM [t(186) = -1.257, p = 0.2102, *d* = -0.092]. Our main results in Fig. 2 are thus not explained by differences in inter-item repulsion.

Supplementary Analysis 2

Testing x Cueing interaction in Exp. 2 is robust to logit transformation. Upon reviewer suggestion, to examine the robustness of this critical interaction effect, we conducted a follow-up analysis using a logit transformation of the LTM data (see also Labaronne et al., 2023, Wagenmakers, 2012). Specifically, we transformed the LTM error data (Fig. 3c) into an "accuracy" value between 0 and 1 [$a = (180 - error)/180$] and applied the logit transformation $log(\frac{p}{1-p})$. After this transformation, the interaction effect remained significant, [F(1,88) = 11.385, p < 0.01, η2 = 0.011], corroborating that the effect was not an artifact of the data's original scale.
