# Supplementary material for: Long-term effects of working memory retrieval from prioritized and deprioritized states
Source: Commun Psychol. 2026 Jan 28;4:32. doi: 10.1038/s44271-026-00399-7 (PMC12913606; doi:10.1038/s44271-026-00399-7)
Supplement: Supplementary file 3 — Reporting summary [file 44271_2026_399_MOESM3_ESM.pdf]

nature portfolio

Corresponding author(s): COMMSPSYCHOL-24-0709-T

Last updated by author(s): 14.12.2024

Reporting Summary

Nature Portfolio wishes to improve the reproducibility of the work that we publish. This form provides structure for consistency and transparency in reporting. For further information on Nature Portfolio policies, see our Editorial Policies and the Editorial Policy Checklist.

Statistics

For all statistical analyses, confirm that the following items are present in the figure legend, table legend, main text, or Methods section.

n/a	Confirmed	
☐	☒	The exact sample size (*n*) for each experimental group/condition, given as a discrete number and unit of measurement
☐	☒	A statement on whether measurements were taken from distinct samples or whether the same sample was measured repeatedly
☐	☒	The statistical test(s) used AND whether they are one- or two-sided<br>*Only common tests should be described solely by name; describe more complex techniques in the Methods section.*
☐	☒	A description of all covariates tested
☐	☒	A description of any assumptions or corrections, such as tests of normality and adjustment for multiple comparisons
☐	☒	A full description of the statistical parameters including central tendency (e.g. means) or other basic estimates (e.g. regression coefficient) AND variation (e.g. standard deviation) or associated estimates of uncertainty (e.g. confidence intervals)
☐	☒	For null hypothesis testing, the test statistic (e.g. *F*, *t*, *r*) with confidence intervals, effect sizes, degrees of freedom and *P* value noted<br>*Give P values as exact values whenever suitable.*
☒	☐	For Bayesian analysis, information on the choice of priors and Markov chain Monte Carlo settings
☐	☒	For hierarchical and complex designs, identification of the appropriate level for tests and full reporting of outcomes
☐	☒	Estimates of effect sizes (e.g. Cohen's *d*, Pearson's *r*), indicating how they were calculated

Our web collection on statistics for biologists contains articles on many of the points above.

Software and code

Policy information about availability of computer code

Data collection	All experiment scripts are designed using the Psychopy library for creating experiments and were translated to java script to be run online. We use several custom libraries and dependencies that are not included in the Psychopy source code. These can be found in our code documentation on GitHub.
Data analysis	Data analysis code was written in R and Python. The analysis code and experiment code are available on GitHub and archived on Zenodo: DOI: 10.5281/zenodo.13867139 and DOI: 10.5281/zenodo.13867798

For manuscripts utilizing custom algorithms or software that are central to the research but not yet described in published literature, software must be made available to editors and reviewers. We strongly encourage code deposition in a community repository (e.g. GitHub). See the Nature Portfolio guidelines for submitting code & software for further information.

Data

Policy information about availability of data

All manuscripts must include a data availability statement. This statement should provide the following information, where applicable:

- Accession codes, unique identifiers, or web links for publicly available datasets
- A description of any restrictions on data availability
- For clinical datasets or third party data, please ensure that the statement adheres to our policy

The data that support the findings of this study are openly available on GIN: DOI: 10.12751/g-node.3p3ryv

Human research participants

Policy information about studies involving human research participants and Sex and Gender in Research.

Reporting on sex and gender

We aimed to recruit a balanced sample of male and female participants for our study using the Prolific platform. Participants were asked to report the gender they most identify with as part of the self-reported demographic information.
Due to server glitches during data acquisition, we encountered multiple duplicate participant IDs, which led to missing demographic information for some participants. These server issues did not affect the collection of behavioral data. Missing demographic information is reported alongside the self-reported gender information below.

For Experiment 1, participants reported the following genders: 58 female, 130 male, and demographic information was missing for 11 participants (n = 199). After excluding participants based on pre-defined criteria, 55 female and 121 male participants remained for analysis, with demographic information missing for 11 participants.

For Experiment 2, participants reported the following genders: 46 female, 42 male, 1 diverse, and demographic information was missing for 12 participants (n = 101). For the final analysis, 41 female, 42 male, and 1 diverse participant remained, with demographic information missing for 5 participants.

For Experiment 3, participants reported the following genders: 44 female, 100 male, 1 diverse, and demographic information was missing for 10 participants (n = 145). After exclusions, 27 female and 50 male participants were included in the analysis, with demographic information missing for 30 participants (n = 107).

Sex and gender were not considered in the design of the study, and no analyses were conducted based on sex or gender.

Population characteristics

The mean age of participants included in the analysis for each experiment was as follows:
Experiment 1: 27.2 years
Experiment 2: 25.0 years
Experiment 3: 27.4 years
No other covariates were reported in this study.

Recruitment

The eligibility criteria were that participants had to be
744 between 18 and 35 years old, fluent in English, have a normal or corrected-to-normal
745 vision, and have a minimum approval rate of 95% on Prolific.

Ethics oversight

The experiment was approved by the Internal Review Board (IRB) of the Max
754 Planck Institute for Human Development.

Note that full information on the approval of the study protocol must also be provided in the manuscript.

Field-specific reporting

Please select the one below that is the best fit for your research. If you are not sure, read the appropriate sections before making your selection.

☐ Life sciences ☒ Behavioural & social sciences ☐ Ecological, evolutionary & environmental sciences

For a reference copy of the document with all sections, see nature.com/documents/nr-reporting-summary-flat.pdf

Behavioural & social sciences study design

All studies must disclose on these points even when the disclosure is negative.

Study description

This study was a quantitative cross-sectional experiment series.

Research sample

Participants were selected based on the inclusion criteria reported above. Given that Prolific recruits participants worldwide, the sample likely comprised individuals with mixed cultural backgrounds.

Sampling strategy

Prolific's automated system randomly assigned eligible participants to the study, ensuring a broad and unbiased sampling of individuals.

Data collection

Data was collected using the institute servers of the Max Planck Institute for Human Development. The study was an online experiment series in which participants were recruited via the Prolific platform. Participants were first provided with introductory content and consent forms hosted on Qualtrics. They then conducted the behavioral experiment on their personal computers via the internet, ensuring accessibility and convenience for participants worldwide.

Timing

The design and preparation for Experiment 1 were completed prior to data collection, which began in September 2022. Following the

analysis of Experiment 1, the design of Experiment 2 was completed, and data collection started in March 2023. Similarly, after analyzing Experiment 2, the design of Experiment 3 was finalized, and data collection began in July 2023.

Data exclusions

Participants were excluded based on pre-defined criteria. Partial payments were made if participants did not complete the experiment due to technical issues. Exclusions were applied as follows for each experiment:

Experiment 1
Technical issues (n = 4), failed attention checks (n = 5), or early termination by the participant (n = 2). One participant (n = 1) was excluded post-experimentally for failing to perform significantly above chance in the working memory (WM) task ($p < 0.05$, t-test against 90° angular error, one-tailed). After applying these exclusions, a total of n = 187 participants remained for analysis (see section above on participant gender information).

Experiment 2
For this experiment, data was not saved for n = 4 participants due to technical problems. Additional exclusions included participants who failed attention checks (n = 2), used paper and pencil to solve the task (n = 1), started the experiment more than once (n = 1), did not enter any data (n = 3), or completed the task but experienced other technical issues (n = 1). After these exclusions, a total of n = 89 participants remained for analysis.

Experiment 3
In this experiment, n = 5 participants were excluded due to failed attention checks, and n = 5 participants were excluded due to technical problems. Among the remaining participants, n = 38 were excluded post-experimentally for failing to perform above chance level in the WM task ($p < 0.05$, Binomial test against 60% correct responses, one-tailed). After these exclusions, a total of n = 107 participants remained for analysis.

Non-participation

Stated in data exclusion section above.

Randomization

Participants were recruited through the Prolific platform, which selects participants randomly while tracking demographic variables (gender). By monitoring this gender covariate, we aimed to recruit a balanced sample of male and female participants for this study. This was the only covariate that was tracked during recruitment.

Reporting for specific materials, systems and methods

We require information from authors about some types of materials, experimental systems and methods used in many studies. Here, indicate whether each material, system or method listed is relevant to your study. If you are not sure if a list item applies to your research, read the appropriate section before selecting a response.

Materials & experimental systems

n/a	Involved in the study	
☒	☐	Antibodies
☒	☐	Eukaryotic cell lines
☒	☐	Palaeontology and archaeology
☒	☐	Animals and other organisms
☒	☐	Clinical data
☒	☐	Dual use research of concern

Methods

n/a	Involved in the study	
☒	☐	ChIP-seq
☒	☐	Flow cytometry
☒	☐	MRI-based neuroimaging
